# Behavioral features in Williams Beuren syndrome: A Tunisian Cohort study

**DOI:** 10.1192/j.eurpsy.2022.1755

**Published:** 2022-09-01

**Authors:** N. Bouayed Abdelmoula, F. Abid, S. Sellami, B. Abdelmoula

**Affiliations:** Medical University of Sfax, Genomics Of Signalopathies At The Service Of Medicine, Sfax, Tunisia

**Keywords:** Williams Beuren syndrome, genome-phenome databases

## Abstract

**Introduction:**

The low prevalence of some genetic neuro-developmental syndromes associated to psychiatric disorders requests to be integrated in human genome-phenome databases from which pleiotropy can be compiled from by systematic integration of phenotypes associated with genetic loci using phenomic inference tools. Williams-Beuren syndrome (WBS) is a neurodevelopmental disorder related to elastin gene at 7q11.23. Anxiety, depression and attention problems are the main behavioral problems found in WBS with no gender differences. Significant differences between cohorts are reported in particular regarding somatic complaints and aggressive behavior.

**Objectives:**

Here, we report a Tunisian cohort of WBS patients for whom clinical behavioral phenotypes as well as genetic features are detailed.

**Methods:**

Sixteen patients from Sfax, Tunisia were referred for genetic assessment due to a suspected WBS syndrome.

**Results:**

Genetic evaluation using fluorescent in situ hybridization confirmed 7q11.23 microdeletion in only eight patients. Comparison of detailed behavioral phenotypes revealed differences between age groups, gender groups and genetic groups. Anxiety and depression were recorded in the two older male patients and aggressive behavior was recorded in only two boys. The severity of behavioral features were dependent to familial environment and to parental socio-economic and educational levels.

**Conclusions:**

A more complete understanding of phenomic space is critical for elucidating genome-phenome relationships mediating neurodevelopmental disorder associated to psychiatric diseases for assessing and managing psychiatric and behavioral risks in young syndromic children.

**Disclosure:**

No significant relationships.

